# Establishing neuronal polarity: microtubule regulation during neurite initiation

**DOI:** 10.1093/oons/kvac007

**Published:** 2022-05-13

**Authors:** Victoria E Higgs, Raman M Das

**Affiliations:** Division of Molecular and Cellular Function, Faculty of Biology, Medicine and Health, University of Manchester, Oxford Road, Manchester M13 9PT, UK; Division of Molecular and Cellular Function, Faculty of Biology, Medicine and Health, University of Manchester, Oxford Road, Manchester M13 9PT, UK

## Abstract

The initiation of nascent projections, or neurites, from the neuronal cell body is the first stage in the formation of axons and dendrites, and thus a critical step in the establishment of neuronal architecture and nervous system development. Neurite formation relies on the polarized remodelling of microtubules, which dynamically direct and reinforce cell shape, and provide tracks for cargo transport and force generation. Within neurons, microtubule behaviour and structure are tightly controlled by an array of regulatory factors. Although microtubule regulation in the later stages of axon development is relatively well understood, how microtubules are regulated during neurite initiation is rarely examined. Here, we discuss how factors that direct microtubule growth, remodelling, stability and positioning influence neurite formation. In addition, we consider microtubule organization by the centrosome and modulation by the actin and intermediate filament networks to provide an up-to-date picture of this vital stage in neuronal development.

## INTRODUCTION

Neurons are one of the most strikingly polarized cell types; their distinct axons and dendrites constitute specialized subcellular regions essential for communication in the nervous system (McCaffrey and Macara, 2012; Campanale et al., 2017). Neuron polarization encompasses the establishment of this architectural and molecular asymmetry in neuronal differentiation (Yogev and Shen, 2017). During development, new-born neurons undergo stepwise morphological and molecular changes as they initiate nascent neurites, thick projections that eventually mature into axons, which transmit action potentials, or dendrites, which receive inputs (Flynn, 2013; Sainath and Gallo, 2015). Neurite initiation constitutes a critical cellular transformation involving coordination of the cytoskeleton, membrane dynamics and intracellular transport. Central to these events are microtubules, a network of dynamic cytoskeletal filaments abundant in neurons, which provide reinforcement, dictate shape and direct trafficking within cells.

Neurite initiation is the critical first step in establishing neuronal connectivity. In the developing embryo, neurite initiation occurs with precise orientation and timing, dependent on neuronal subtype. This process is controlled by a plethora of regulators and directed by signals from surrounding tissue (Flynn, 2013; Sainath and Gallo, 2015). Neurite malformation has been reported in models of autism spectrum disorder (ASD) (Van Maldergem et al., 2013; Nguyen et al., 2018), schizophrenia (Miyoshi et al., 2003; Ma et al., 2011), lissencephaly (Shahsavani et al., 2018) and Phelan McDermid syndrome (Yi et al., 2016), suggesting that dysregulation of this event contributes to the circuit formation abnormalities thought to underlie these conditions.

Microtubule remodelling underpins cellular polarization and consequently microtubules are a key target of neuronal polarity signalling (Arimura and Kaibuchi, 2007; Kapitein and Hoogenraad, 2015). Many neurodevelopmental disorders are associated with microtubule regulatory proteins (Lasser et al., 2018), the diverse group of factors that fine tune microtubule growth, stability, localization, shape and interactions with other cellular components.

 Although microtubule regulation has been well characterized in later stages of neuronal differentiation, microtubule regulation during neurite initiation is less well understood and overviews of the subject are lacking. In this review, we draw from studies in a variety of contexts to discuss how microtubules are regulated in neurite initiation, with a particular focus on vertebrate studies. Highlighting recent advances in the field, we examine how microtubule-interacting proteins, nucleation sites, cytoskeletal crosstalk and extracellular signalling cooperate to regulate this critical process in nervous system development.

## STUDYING NEURONAL POLARITY AND MORPHOGENESIS

During differentiation, new-born neurons derived from progenitors must transform their morphology to establish complex sprawling neuronal architecture. In vertebrates, central nervous system (CNS) neurons originate from the neuroepithelium of the neural tube (Götz and Huttner, 2005; Paridaen and Huttner, 2014). This tissue initially consists of a single layer of polarized multipotent neuroepithelial cells stretched across the width of the tissue, contacting apical and basal surfaces. As development advances, postmitotic neurons are generated either directly from neuroepithelial cells or, in some cases, such as in the cerebral cortex, indirectly via intermediate cell types. In committed neurons, morphogenesis proceeds in a highly stereotyped spatiotemporal manner depending on neuronal subtype and context (Arimura and Kaibuchi, 2007; Namba et al., 2015; Yogev and Shen, 2017). For example, in the rodent cerebral cortex, neurons go through a multipolar phase, extending multiple neurites before becoming bipolar, with a trailing process that will become the axon, and a leading process that will become a dendrite (Yogev and Shen, 2017). In contrast, chick spinal cord neurons, which directly differentiate from elongated neuroepithelial cells, detach from the apical surface, and withdraw their apical process to delaminate from the proliferative ventricular zone (Das and Storey, 2014; Kasioulis and Storey, 2018). The axonal neurite is then the first process extended from the cell body, emerging in the direction of the target (Kasioulis and Storey, 2018; Toro-Tapia and Das, 2020).

The technical challenges of studying the formation of complex 3D neuronal structures within tissue have meant that intracellular processes such as cytoskeletal regulation are often studied in 2D culture. Classically, dissociated plated neurons, usually from the rodent hippocampus, pass through a well-characterized set of morphological stages, first extending filopodia and lamellipodia (stage 1), which resolve into multiple non-specific neurites (stage 2) before one is specified as an axon and rapidly elongates (stage 3), following which the others become dendrites (stage 4) and the processes mature (Dotti, Sullivan and Banker, 1988). As evidenced by the propensity for the first neurite to be specified as the axon and to define where the second neurite emerges from (Lefcort and Bentley, 1989; Zmuda and Rivas, 1998; De Anda et al., 2005, 2008, 2010), neurite fate might already be sealed before stage 3. The establishment of the first neurite can thus be considered the initiation of neuronal polarization in vitro (Cáceres et al., 2012; Flynn, 2013).

Although primary neuron culture has facilitated identification of many microtubule regulators important for establishment and maintenance of neuronal polarity, neurons in this context lack the rich signalling milieu of the embryo and consequently the mechanisms directing neurite formation within developing tissue are only partially understood. Indeed, one study elegantly demonstrated that the same neuronal subtype polarizes differently in vitro and in vivo—retinal ganglion cells in vivo bypass the multipolar morphology they display in culture (Zolessi et al., 2006). Furthermore, dissociated cultures could also include neurons that have polarized in vivo but lost their projections during dissociation, thus retaining aspects of polarity (Barnes and Polleux, 2009). De-novo neurite formation is regularly studied in growth-stimulated neuroblastoma (e.g. Neuro2A) or PC-12 cells, which form non-specific neurites, but these cells are likely to substantially differ from neurons (Sainath and Gallo, 2015). This issue has recently been circumvented through the use of human pluripotent stem cell-derived neurons, which facilitate monitoring of de-novo neurite initiation during human neuronal differentiation (Lindhout et al., 2021).

In vivo, the timing, order and orientation of neurite formation are highly regulated. For example, in neurons of the spinal cord that directly extend an axon and do not exhibit a multipolar phase, the axonal neurite is initiated with stereotyped orientation (Hadjivasiliou et al., 2019; Moore et al., 2020; Toro-Tapia and Das, 2020). In some neurons, neurite initiation sites might be defined at the progenitor stage. For example, after cytokinesis, *Drosophila* sensory neurons retain apical polarity components at their apical pole and accumulate PIP2 and then α-catenin, DE-Cadherin and Bazooka/Par3 at the site before neurite initiation (Pollarolo et al., 2011). Likewise, zebrafish bipolar cells and retinal ganglion cells can develop their axons directly from a basal process (Zolessi et al., 2006; Yogev and Shen, 2017), and chick dorsal root ganglion neurons inherit sites of neurite formation from their neural crest cell ancestors (Boubakar et al., 2017). For neurons that need to re-establish polarity during differentiation, external cues are key. For example, spinal cord neurons lose their apical polarity components during apical abscission, demonstrated by the absence of Par complex proteins aPKC and Par3 from the withdrawing apical process (Das and Storey, 2012, 2014), and then initiate the axonal neurite along an adjusted axis (Toro-Tapia and Das, 2020). In such cases, polarization is heavily guided by cues from the surrounding tissue. Indeed, contact cues such as cadherins (Gärtner et al., 2012) and diffusible cues such as netrins and Shh guide neurite initiation and extension (Adler et al., 2006; Toro-Tapia and Das, 2020). This evidence highlights the importance of the embryonic environment in directing neuronal polarity.

## MICROTUBULES IN NEURITE INITIATION

### Microtubule dynamics

Microtubules are tubular polymers formed of α-tubulin–β-tubulin heterodimers (Etienne-Manneville, 2010; Hawkins et al., 2010). These subunits polymerize into protofilaments, 11–15 of which associate in parallel to make a sheet, which is sealed to create a tube, mainly by lateral interaction of alike monomers. Their tubular lattice structure makes microtubules inflexible along their lengths, with most polymerization and depolymerization occurring at the ends. Tubulin heterodimers face in the same direction within the filament, giving microtubules polarity, α-tubulin exposed at the so-called minus end, and β-tubulin exposed at the so-called plus end. This polarity facilitates selective activity of motor proteins, which track along microtubules to transport cargo (Prokop, 2013) or generate force (Hawkins et al., 2010).

The dynamic property of microtubules enables rapid remodelling during cell state changes and relies upon the inherent guanosine triphosphate (GTP)ase activity of β-tubulin (Etienne-Manneville, 2010). In each tubulin dimer, the α-subunit is constitutively associated with a GTP, whereas β-tubulin alternately associates with guanosine disphosphate (GDP) and GTP. Under physiological conditions, GTP-bound β-tubulin favours polymerization and GDP-bound tubulin favours depolymerization. Microtubules display dynamic instability, in which periods of slow growth and rapid shrinking occur, enabling reorganization of the network. Growing microtubules have a cap of GTP-bound subunits at the plus end. Bound GTP is hydrolysed to GDP after dimer incorporation into the lattice. When GTP hydrolysis occurs faster than subunit addition, the GTP cap is depleted, and the microtubule undergoes catastrophe and begins depolymerizing. Conversely, rescue of growth occurs when GTP-bound tubulin is replenished at the plus end. Microtubule network remodelling is central to the establishment of cellular polarity, enabling transformation of cell structure and rigidity, and the asymmetric transport of cellular components.

### Microtubule remodelling in neurite formation

The mature neuron has an asymmetric network of stable microtubules (Yogev and Shen, 2017). Axons and dendrites are packed with stable parallel bundles of microtubules with characteristic orientation, in vertebrates, facing plus-end out in axons, and a mixture of plus-end out and minus-end out in dendrites (Conde and Cáceres, 2009; Leterrier et al., 2017).

A neurite is a microtubule-rich neuronal protrusion that elongates to at least the length of the cell body (Dehmelt and Halpain, 2004), and the term is generally used to refer to early axons and dendrites regardless of their fate. The neurite shaft contains mixed-orientation microtubule bundles (Miller and Suter, 2018). At the growing tip is a specialized sensory region called the growth cone. First observed by Santiago Ramón y Cajal in the late 1800s, he correctly postulated the filament-rich expanded tip to be a dynamic structure necessary for orienting process growth (Tamariz and Varela-Echavarría, 2015). The peripheral domain of the growth cone consists of actin-rich filopodia and lamellipodia (Conde and Cáceres, 2009; Lowery and Van Vactor, 2009). Microtubules dominate the central domain; their constant remodelling necessary for process extension. At the transition zone between these domains, the depolymerizing ends of actin filaments interact with microtubule plus ends, and contractile actin arcs form a barrier that restricts microtubule protrusion into the peripheral zone.

**Figure 1:**
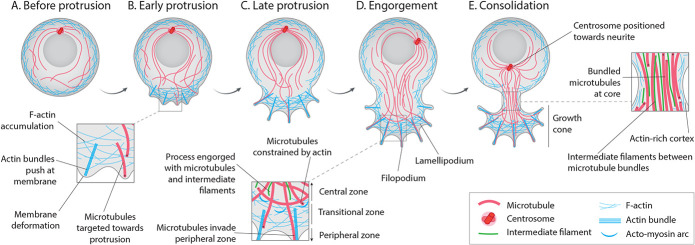
Stages of neurite initiation. (**A**) Before a neurite is initiated, microtubules are nucleated at the centrosome and arranged loosely within the cell. (**B**) In the early protrusion phase, F-actin accumulates at the site of the future neurite and microtubules are targeted towards the protrusion. The plasma membrane is deformed in an F-actin-dependent manner, although microtubule-generated force may also contribute. (**C**) The protrusion develops into an actin-rich lamellipodium with filopodia. (**D**) In the engorgement phase, microtubules and intermediate filaments advance into the protrusion as the growth cone forms. Most microtubules are constrained to the central zone by F-actin and contractile acto-myosin arcs in the transitional zone. Some microtubules penetrate the peripheral zone, guided by F-actin bundles. (**E**) In the consolidation phase, the plasma membrane proximal to the cell body subsides and cytoskeletal filaments condense, resulting in a dense core of microtubules and intermediate filaments. The centrosome is typically positioned ahead of the nascent neurite. For simplicity, intermediate filaments are only shown in zoomed sections. F-actin=filamentous actin.

Neurite formation has been described to occur in three stages (Fig. 1): protrusion, engorgement and consolidation (Flynn, 2013) on the basis of earlier characterization of axon elongation (Goldberg and Burmeister, 1986). During protrusion, rearrangement and polymerization of actin filaments pushes outward to deform the plasma membrane. This initial stage is heavily actin dependent and can proceed in the presence of microtubule depolymerizing or polymerization-inhibiting drugs (Smith, 1994b; Moore et al., 2020). In some dissociated neurons, a large lamellipodium is formed around the cell border, which then subdivides into multiple nascent growth cones (Yu et al., 2001; Dehmelt et al., 2003; Flynn et al., 2012). During engorgement, microtubules, intermediate filaments (IFs), vesicles and organelles move into the process (Smith, 1994a; Flynn, 2013; Zhang et al., 2016). Following this, during consolidation, the proximal membrane subsides, and cytoskeletal elements condense, bundling the microtubules into a dense cylindrical core (Tang and Goldberg, 2000; Yu et al., 2001; Dehmelt and Halpain, 2004; Flynn et al., 2012). An alternative mode of neurite formation has also been described in dissociated neurons (Smith, 1994a; Dent et al., 2007; Flynn et al., 2012; Sainath and Gallo, 2015), whereby a filopodium swells with microtubule bundles and acquires a growth cone at its tip.

Microtubule depolymerization is associated with reduced numbers of neurites in dissociated neurons (Dent et al., 2007; Flynn et al., 2012; Zhang et al., 2016; Verstraelen et al., 2017), suggesting that neurite formation is dependent on microtubules. In spinal neurons of the *Xenopus* embryo, nascent axon growth is restricted to a short stub when microtubules are depolymerized (Moore et al., 2020). Although well characterized during the later stages of neurite and axon formation, microtubule rearrangements during neurite formation have not been fully described. Cytoskeletal organization and regulation in the cell body before appearance of neurites is especially poorly understood (Sainath and Gallo, 2015). However, actin aggregates form at future initiation sites (Zhang et al., 2016), and asymmetric microtubule activity has been demonstrated in stage 1–2 dissociated neurons, with microtubules growing towards one side of the morphologically unpolarized cell (Gärtner et al., 2014) and selectively advancing into future neurite-forming regions of the periphery (Flynn et al., 2012). In Neuro2A cells without processes, growing microtubule plus ends concentrate in discrete areas of the cell periphery, suggesting this might precede neurite initiation (Morrison et al., 2002). Recent electron microscopy analysis revealed that microtubule plus tips can provide a platform for nucleation of branched filamentous actin (F-actin) networks towards the cell periphery (Efimova et al., 2020), which might aid in the protrusion phase of initiation.

Microtubules are a core feature of the engorgement and consolidation phases, likely performing several critical roles for the nascent neurite. As the most rigid cytoskeletal element, microtubules reinforce cell structure, stabilize protrusions and cooperate with other filaments to generate the tensile forces required for shape change (Hawkins et al., 2010). Additionally, microtubules direct trafficking within neurons (Kapitein and Hoogenraad, 2011). The microtubule bundles formed during consolidation are an essential feature of axon biology, forming a structural backbone along which cargo can be moved (Hahn et al., 2019). In this way, microtubules facilitate asymmetric transport of the cytoskeletal components, adhesion molecules, plasma membrane and signalling proteins necessary for neurite formation (Dehmelt and Halpain, 2004; Pfenninger, 2009).

In multipolar phase neurons in vitro (Cáceres et al., 2012) and in vivo (Tabata and Nakajima, 2002; Noctor et al., 2004), the non-specific neurites that initially form are highly dynamic, retracting and extending before one undergoes axon specification, leading to rapid elongation. Neurite initiation and axon specification likely share numerous mechanisms; however, microtubule regulation at these stages is not identical. For example, microtubule stabilization with low doses of the drug paclitaxel can induce axon specification (Witte and Bradke, 2008) but not initiation of neurites (Flynn et al., 2012) in culture. Additionally, axon specification is associated with a microtubule polarity sorting event. Microtubules in non-specific neurites initially have mixed polarity, which resolves into plus-end-out orientation after axon specification by the elimination of minus-end-out filaments (Yau et al., 2016; Rao and Baas, 2018). Whether the events underlying axon specification apply to neurons that do not exhibit a multipolar phase is unclear. Directly formed spinal cord axons steadily persist growth after initiation without the cycles of protrusion and retraction associated with non-specific neurites (Moore et al., 2020; Toro-Tapia and Das, 2020). Therefore, although studies of axon specification are informative about neurite initiation, the topic, including microtubule polarity sorting, will not be discussed in detail here, and the reader is referred to several excellent reviews (Barnes and Polleux, 2009; Lalli, 2012; van Beuningen and Hoogenraad, 2016; Rao and Baas, 2018).

## REGULATION OF MICROTUBULES IN NEURITE INITIATION

### Neuronal polarity signalling and microtubules

Cellular polarization is usually achieved by asymmetric distribution of intracellular components (McCaffrey and Macara, 2012). Polarization broadly occurs in three stages. First, symmetry is broken either by an environmental cue or stochastically. Second, intracellular signalling results in establishment of spatial organization. Finally, polarity is amplified and maintained by feedback mechanisms. Although the regulatory pathways are not fully understood, neurite initiation likely involves the interaction of many signalling pathways that converge on the cytoskeleton (Sainath and Gallo, 2015). For example, Rho GTPases Cdc42, Rac and RhoA are key upstream regulators of microtubules and actin thought to be central to neurite formation by extracellular factors. In the proposed pathway, neurotrophin-mediated (e.g. nerve growth factor (NGF) or brain-derived neurotrophic factor (BDNF)) activation of Trk receptors activates PI3K, leading to accumulation of PIP3 at the cell periphery and activation of GTPases Cdc42 and Rac, which encourage neurite formation, and a decrease in RhoA signalling, which negatively regulates neurite formation (Sainath and Gallo, 2015). Positive and negative feedback loops are suggested to create discrete domains of PIP3 and Rac activity that form neurite initiation sites (Nakamura et al., 2008). Rho GTPases are also key downstream targets for other regulators of neurite initiation, such as netrin (Serafini et al., 1996; Charron et al., 2003; Boyer and Gupton, 2018), N-cadherin (Gärtner et al., 2012; Gärtner et al., 2015), fibroblast growth factor (FGF) (Togari et al., 1985; Harada et al., 2005) and laminin (Lander et al., 1985; Weston et al., 2000). Polarity signalling likely coordinates the many factors involved in microtubule remodelling and other processes in neuronal differentiation, ensuring they act in concert.

### Microtubule-interacting proteins

**Figure 2:**
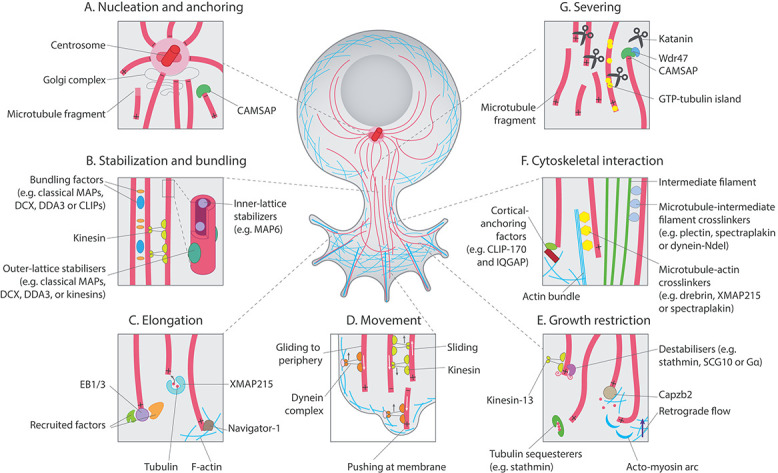
Possible microtubule regulatory mechanisms in neurite initiation. Microtubules are tightly regulated by a variety of factors during neuronal development. (**A**) The centrosome and Golgi complex act as microtubule-organizing centres, nucleating and anchoring microtubules. Microtubule fragments can also seed nucleation, and factors such as CAMSAPs can stabilize minus ends. (**B**) Stabilization of microtubules is influenced by factors that interact with the outer or inner microtubule lattice. Outer-lattice binding proteins can also contribute to bundling of filaments, along with kinesin motor proteins. (**C**) Plus-tip binding proteins aid in microtubule elongation. Here, end-binding proteins enhance polymerization and recruit other regulators. Delivery of tubulin dimers by factors such as XMAP215 assists in polymerization and molecules such as Navigator-1 may help prevent depolymerization in actin-rich regions. (**D**) Movement of microtubules by motor proteins could facilitate neurite initiation in several ways. Kinesins enable sliding of microtubules against one another to generate force and dynein-based complexes may assist in gliding microtubules towards the periphery or pushing them end on into the plasma membrane via interactions with the cortex. (**E**) Mechanisms also exist to restrict microtubule growth or advance. Destabilizing factors such as kinesin-13 or stathmins enhance depolymerization, which might assist microtubule dynamics and remodelling. Polymerization may also be inhibited by actin-binding protein Capzb2 or factors that sequester tubulin. Actin can also restrict microtubules by retrograde flow or formation of contractile acto-myosin arcs. (**F**) F-actin bundles, however, can guide microtubule growth, an action facilitated by crosslinker proteins. Microtubules can also be anchored at the actin cortex by factors such as CLIP170 and IQGAP. Intermediate filaments might act to space microtubules and template their growth via crosslinking proteins. (**G**) Finally, severing proteins, such as katanin, fragment microtubules to facilitate transport and generate new plus ends. Their action can also generate GTP-tubulin islands, which may promote microtubule rescue and regulate factor binding. Minus ends may be protected from severing by CAMSAPs and Wdr47. F-actin=filamentous actin. GTP=guanosine triphosphate.

Within neurons, microtubule dynamics are enhanced by stabilizing and destabilizing factors, along with severing proteins and motor proteins that cooperate to accelerate and localize network remodelling (Conde and Cáceres, 2009; Voelzmann et al., 2016). Hundreds of such proteins have been associated with neurite and axon formation; many likely have overlapping roles to ensure this vital developmental event proceeds in different scenarios (Arimura and Kaibuchi, 2007; Arens et al., 2013; Flynn, 2013; Kapitein and Hoogenraad, 2015; Menon and Gupton, 2016). Here, we summarize the key factors likely to be relevant to neurite initiation (Fig. 2).

#### Stabilizing and bundling proteins

Neurons contain a higher proportion of stable microtubules than dividing cells (Baas et al., 2016). During differentiation, long-lived microtubules are required to create force, reinforce structure, and maintain trafficking (Flynn, 2013). In dissociated neurons, microtubules in the neurite shaft are stable and bundled, stable microtubules increasing as the neurite matures into an axon (Witte et al., 2008). A variety of mechanisms can increase microtubule stability during neurite formation with various outcomes on cell behaviour. Additionally, there is functional overlap between various factors, emphasizing the range of mechanisms differentiating neurons can draw on to fine tune microtubule stability.

The MAP2/Tau and MAP1A/MAP1B families of microtubule-associated proteins (MAPs) are perhaps the most well-known microtubule stabilizing proteins (Flynn, 2013; Baas et al., 2016). These so-called classical or structural MAPs bind and stabilize the microtubule lattice, enhance bundle formation and assist in the association of other proteins with microtubules. MAP2C and MAP1B are particularly relevant to neurite initiation. The MAP2/Tau family laterally associate with and stabilize microtubules by preventing catastrophe and promoting rescue (Dehmelt and Halpain, 2004). Overexpression of MAP2C triggers neurite initiation in Neuro2A cells and hippocampal neurons via accumulation and bundling of microtubules (Dehmelt et al., 2003, 2006). Conversely, disruption of the MAP2C microtubule-binding domain prevents neurite initiation (Dehmelt et al., 2003). Furthermore, MAP2C overexpression in non-neuronal cells results in formation of extended microtubule bundles (Weisshaar et al., 1992) and also neurite formation if actin is concurrently depolymerized (Edson et al., 1993). Whereas MAP2C appears to be important for neurite initiation, Tau seems to be more important later in development, since antisense oligonucleotides decrease axon extension but do not decrease neurite formation (Cáceres et al., 1991).

MAP1B is another microtubule-stabilizing protein (Villarroel-Campos and Gonzalez-Billault, 2014), which is proposed to functionally overlap with MAP2 (Teng et al., 2001). In PC-12 cells, MAP1B antisense oligonucleotides inhibit neurite formation in a reversible manner (Brugg et al., 1993). Additionally, knockdown in neuroblastoma cells interferes with bundle formation in neurites (Feltrin et al., 2012). However, cultured neurons from a mouse line low in MAP1B are able to develop neurites, although axon formation is inhibited (Gonzalez-Billault et al., 2001; Gonzalez-Billault et al., 2002). Functional redundancy or compensation might underlie this discrepancy. For example, hippocampal neurons with low MAP1B have more MAP2 in the axon (Gonzalez-Billault et al., 2001). Overall, further studies are required to uncover the precise role of MAP1B during neurite initiation.

Proteins that accumulate at microtubule plus tips can also regulate stability and bundling. Doublecortin associates along microtubules in the grooves between protofilaments (Baas et al., 2016) but is especially enriched at the plus tip (Reiner, 2013). In the consolidation phase of axon outgrowth, doublecortin interacts with microtubules to bundle and stabilize them (Bielas et al., 2007). Its perturbation is associated with defects in axon formation, including disordered axonal microtubules. Excess doublecortin in Neuro2A cells, achieved by overexpression of its positive regulator 14-3-3-ε, results in reduced microtubule invasion into lamellipodia and filopodia and reduced formation of neurites, a phenotype that can be rescued by doublecortin knockdown (Cornell et al., 2016). Conversely, 14-3-3-ε deficiency enhances neurite formation in the mouse cortex. This evidence suggests that doublecortin also functions in the engorgement phase of neurite initiation.

The vertebrate neuronal protein MAP6 (formerly known as STOP) confers stability to microtubules by binding the inner lattice (Cuvellier et al., 2021). In cultured PC-12 cells, injection of anti-MAP6 antibodies or antisense oligonucleotides reduced neurite formation during differentiation (Guillaud et al., 1998), suggesting that MAP6-mediated microtubule stabilization is required for this process. However, experiments on cultured hippocampal neurons showed MAP6 was only enriched in the proximal specified axon and not earlier neurites (Tortosa et al., 2017), thus its role in neurite initiation in neurons is unclear.

Microtubule stabilizers can also negatively regulate neurite formation. For example, DDA3 is a microtubule stabilizing and bundling protein that interacts with the microtubule plus end (Hsieh et al., 2012; Zhang et al., 2013). Depletion of DDA3 results in spontaneous neurite formation in unstimulated Neuro2A cells and increases neurite and axon formation in cultured hippocampal neurons (Hsieh et al., 2012). Likewise, centrosomal protein centlein has been identified as a microtubule stabilizer that can reduce neurite formation (Jing et al., 2016).

#### Motor proteins

Microtubule stabilization with low-dose paclitaxel enhances axon growth in dissociated hippocampal neurons by enhancing polymerization at neurite tips (Witte et al., 2008; Flynn et al., 2012). However, the same level of paclitaxel does not increase neurite formation in cultured neurons (Flynn et al., 2012). These studies suggest that neurite initiation is reliant on mechanisms additional to microtubule polymerization.

Motor proteins kinesin and dynein track along microtubules, generally towards the plus end or minus end respectively, rearranging their cargos within the cell (Hirokawa et al., 2010). Among this cargo, motor proteins move other microtubules, enabling their redistribution and facilitating the generation of force (Lu and Gelfand, 2017). Microtubule sliding involves the movement of short microtubule polymers by motors and drives neurite initiation (Miller and Suter, 2018). Beyond initiation, as the axon elongates, motors are also involved in microtubule polarity sorting, and bulk transport. These topics are covered in recent reviews and will not be detailed here (Miller and Suter, 2018; Rao and Baas, 2018).

Kinesin-1 can bridge microtubules and slide them against one another (Lu and Gelfand, 2017). In *Drosophila* neurons, neurite initiation can proceed via microtubule pushing at the membrane despite actin depolymerization or inhibition of microtubule polymerization; however, reduction of kinesin-1 heavy chain inhibits neurite formation (Lu et al., 2013), suggesting that sliding-based force is important for the early stages of outgrowth. Consistently, kinesin-1 heavy chain knockdown in cultured hippocampal neurons leads to the stunting of neurite growth (Ferreira et al., 1992) and overexpression of kinesins in non-neuronal insect sf9 cells induces the formation of neurites (Sharp et al., 1996; Sharp et al., 1997). Sliding by kinesin-1 requires antiparallel filament organization, suggesting that this orientation is advantageous to early neurite outgrowth (del Castillo et al., 2015). This might explain why all neurites emerge with antiparallel arrays despite axons later acquiring a uniform plus-end-out arrangement.

Kinesins have been shown to mediate a variety of other effects on neuronal microtubules (del Castillo et al., 2019). Kinesin-13 proteins catalyse microtubule depolymerization, which could assist in network remodelling. Knockout of kinesin-13 KIF2A results in supernumerary neurites in dissociated cortical neurons (Ogawa and Hirokawa, 2015) and this protein regulates morphology of dentate granule cells in mice (Homma et al., 2018). In crosslinking microtubules, kinesins can also be involved in microtubule bundling that dampens sliding (del Castillo et al., 2019). Mitotic kinesins can stabilize antiparallel microtubules at the spindle and are an example of mitotic machinery reuse in neurite formation (Rao and Baas, 2018). In particular, kinesin-6 acts at the cell body to limit microtubules that enter the neurite. Reduction of kinesin-6 protein Pavarotti in *Drosophila* neurons with RNA interference results in increased microtubule sliding and neurite numbers, revealing its role as a negative regulator of kinesin-1 mediated sliding (del Castillo et al., 2015).

Dynein has also been implicated in the creation of force in neurite initiation, but without coupling microtubules (Miller and Suter, 2018). In neuroblastoma cells, introduction of dynein-inhibiting antibodies inhibits the formation of neurites and impairs the radial transport of microtubule bundles to the cell periphery (Dehmelt et al., 2006). Dynein-based capture of microtubule plus tips at the cell cortex could facilitate outward pushing of microtubules (Mazel et al., 2014). In support, overexpression of the motor domain of another minus-end directed motor protein, CHO2, in sf9 cells induces formation of neurites with plus-end-out directed microtubules (Sharp et al., 1997). Dynein might create this movement by interacting with lateral surface of microtubules to facilitate a longitudinal gliding mechanism. However, dynein has also been suggested to aid in stabilization of microtubule plus ends at the cortex (Hendricks et al., 2012) and could tether microtubules at an angle to the cell membrane (Gusnowski and Srayko, 2011), which might facilitate end-on force generation.

#### Severing proteins

For microtubules to be transported within the differentiating neuron, they can be fragmented into shorter polymers by the action of severing enzymes, such as katanin and spastin (Conde and Cáceres, 2009; Sharp and Ross, 2012; Kapitein and Hoogenraad, 2015). This also expands the microtubule network by generating more microtubule ends for polymerization. Katanin is highly expressed by neurons in early axonal growth (Karabay et al., 2004), whereas spastin appears to have roles later in axon branching and maintenance (Conde and Cáceres, 2009; Menon and Gupton, 2016). Work in cultured sympathetic neurons showed that while it is present throughout the neuron, katanin is especially active at the centrosome (Ahmad et al., 1999). Injection of an inactivating antibody resulted in reduction of neurite outgrowth and a build-up of microtubules at the centrosome after microtubule depolymerization, suggesting that it releases centrosomal microtubules to facilitate neurite formation. Likewise, expression of a dominant-negative katanin active subunit (P60) inhibits severing and suppresses axon growth (Karabay et al., 2004). Overexpression of wild-type P60 also causes excess severing (Karabay et al., 2004) and reduces the total number of neurites grown (Yu et al., 2005). These studies suggest that katanin activity is finely controlled by other factors in neurite formation. Some cultured neurons appear resistant to the deleterious effect of P60 overexpression on axon outgrowth (Karabay et al., 2004), suggesting that regulatory factors in some neurons can prevent or compensate for excess severing. Indeed, MAP2, which is present in early neurites (Cáceres et al., 1986), inhibits katanin activity in neurons (Qiang et al., 2006). Additionally, Wdr47, which co-operates with minus-end stabilizer CAMSAP2 at minus ends, has recently been identified to protect minus ends from severing by katanin in developing neurons (Buijs et al., 2021). Conversely, acetylation of microtubules is associated with increased sensitivity to katanin (Sudo and Baas, 2010). Katanin and spastin can also amplify microtubule arrays by extracting tubulin dimers from along microtubule shafts (Vemu et al., 2018). GTP-tubulin is spontaneously incorporated into these sites, forming GTP-tubulin islands, which are thought to protect plus ends from depolymerization by promoting rescue events. In hippocampal neurons, GTP islands are selectively abundant in the future axonal neurite and have been proposed to form binding sites for kinesin-1 (Nakata et al., 2011), suggesting that severing could also localize the activity of other microtubule regulators in developing neurons.

#### Regulation at the plus end

Dynamic instability of microtubules enables the neuronal cytoskeleton to rapidly remodel through cycles of growth and shrinkage. In purified tubulin experiments, enhancers of both polymerization and depolymerization are required to recapitulate the high polymerization and catastrophe rates microtubules exhibit in vivo (Kinoshita et al., 2001). In building a neuron, cyclic disassembly and reassembly at microtubule plus ends enables adaptable neurite growth in response to the environment. Thus, the two states are tightly controlled by numerous factors. Reducing microtubule polymerization without causing depolymerization using low-dose nocodazole inhibits neurite formation in cultured neurons (Dent et al., 2007; Flynn et al., 2012). Additionally, high-dose paclitaxel, which leads to the formation of stable microtubule aggregates, also inhibits neurite initiation (Zhang et al., 2016). Likewise, eribulin, a drug that inhibits microtubule dynamics, restricts microtubule protrusion into lamellipodia and inhibits neurite formation in cultured cortical neurons in a dose-dependent fashion (Poobalasingam et al., 2021). These studies suggest that microtubule dynamicity is necessary for neurite formation.

Plus-tip interacting proteins (+TIPs) are a diverse group of proteins that associate with the microtubule plus tip to regulate polymerization, steer microtubule growth and concentrate signalling (Akhmanova and Steinmetz, 2015). +TIPs function in networks, with end-binding proteins (EBs) thought to be core components, providing binding sites for other interactors. Their plus-tip tracking quality makes fluorescent EB fusion proteins especially useful for visualizing growing microtubule ends in live cells (Stepanova et al., 2003). EBs bind multiple GTPs to stabilize bonds between tubulin dimers and promote catastrophe in vitro, potentially by enhancing tubulin GTP hydrolysis. However, in cells, EBs promote microtubule dynamics and elongation, likely due to their interactions with other regulators. RNA-interference-mediated knockdown of EB1 in neuroblastoma cells is associated with decreased microtubule growth rate and distance, and decreased neurite growth (Stepanova et al., 2010). However, EB3 might be more important for neurite initiation. Loss of EB3 but not EB1 function using dominant negative constructs or CRISPR-Cas9 genome editing inhibits neurite formation in rat cortical neurons (Geraldo et al., 2008; Poobalasingam et al., 2021).

CLIP-170 and CLIP-115 are cytoplasmic linker +TIPs, which promote microtubule stability (van de Willige et al., 2016). CLIPs are present in the growth cones of non-specific neurites and enable the protrusion of microtubules into the peripheral zone of the axonal growth cone, suggesting that they could function in neurite formation (Neukirchen and Bradke, 2011). However, decreased tubulin detyrosination (which regulates CLIP binding) in hippocampal neurons reduces CLIP-170 localization to growth cones and is associated with increased neurite growth (Erck et al., 2005), suggesting that precise regulation of CLIP function by other factors may modulate their effect on neurite extension.

The role of +TIPs in neurite formation might be better understood by considering interplay between +TIPs and other microtubule regulators. In neuroblastoma cells, EB1 and EB3 have been found to be regulated by their direct interactor MAP1B, and all three proteins are enriched in neurites (Tortosa et al., 2013). Overexpression of MAP1B sequesters EB1 and EB3 from plus ends and increases speed of microtubule growth. Knockdown of EB2 is associated with an increase in neurite length in neuronally differentiated P19 cells, suggesting it can act as a negative regulator of neurite growth, perhaps due to competition with other EBs for binding sites (Arens et al., 2013). The XMAP215 family of +TIPs are microtubule polymerases that recruit tubulin to plus ends, facilitating polymerization (Akhmanova and Steinmetz, 2015). Their roles in neurite initiation are unclear; however, recent work in cultured *Drosophila* and *Xenopus* neurons has demonstrated cooperation between XMAP215/Msps, Tau, and EB1 to regulate microtubule growth and bundling in axon formation (Hahn et al., 2021). Other +TIPs, such as TACC3 (Erdogan et al., 2017), APC (Zhou et al., 2004) and CLASPs (Hur et al., 2012) have also been implicated in axon development, but their roles in neurite initiation are unclear.

Proteins that bind tubulin dimers can also affect polymerization or depolymerization of microtubules. The stathmin/SCG10 family can sequester tubulin dimers or bind to microtubule plus ends causing destabilization (Grenningloh et al., 2003). SCG10 is a neuron-specific stathmin that is enriched in growth cones. Overexpression of SCG10 in PC-12 cells increases the proportion of cells bearing neurites and the length of these neurites (Riederer et al., 1997). In cultured neurons, precise SCG10 levels are required for proper neurite outgrowth (Morii et al., 2006). Reducing SCG10 activity in cultured hippocampal neurons reduces neurite length, increases the formation of looped microtubule conformations in the growth cone and reduces growth cone motility. In this context, SCG10 is believed to increase catastrophe frequency enabling remodelling (Grenningloh et al., 2003).

Another group of proteins that have been proposed to increase microtubule catastrophe in neuronal differentiation are the α subunits of heterotrimeric G proteins (Gα), which can increase hydrolysis of GTP by tubulin (Wang et al., 1990; Wang and Rasenick, 1991; Roychowdhury et al., 1999). Overexpression of a Gαi1 in COS-1 cells increases the number and length of cellular processes (Chen et al., 2003). Overexpression of an active mutant of Gαs in PC-12 cells increases the proportion of cells with neurites and diminishes the pool of stable microtubules within the cells (Yu et al., 2009), suggesting that activated Gα proteins can increase tubulin depolymerization to enhance neurite outgrowth.

### Microtubule-organizing centres

The growth of a neurite requires expansion of the microtubule network. One way that cells can achieve this is the nucleation of new microtubules from a microtubule-organizing centre (MTOC). Within cells, microtubules require nucleating factors to assemble (Petry and Vale, 2015; Roostalu and Surrey, 2017). In most cell types, nucleation occurs mainly at the centrosome, a membraneless organelle composed of a pair of centrioles surrounded by a matrix of pericentriolar material (PCM) rich in pericentrin, which recruits other centrosomal components (Doxsey, 2001; Wu and Akhmanova, 2017). γ-tubulin, a homolog of α-tubulin and β-tubulin, is considered the key PCM component responsible for nucleating microtubules (Kollman et al., 2011). At the PCM, γ-tubulin associates with other proteins forming the ring-shaped γ-tubulin ring complex (γ-TURC), which facilitates longitudinal attachment of tubulin heterodimers, thereby nucleating de-novo microtubule assembly. γ-TURC thus forms a minus-end cap, anchoring microtubules at the centrosome and stabilizing them by blocking dynamics (Wu and Akhmanova, 2017). This anchoring results in the formation of a characteristic radial array of microtubules originating from the centrosome.

Non-centrosomal microtubule organization is a feature of many differentiated cells, including neurons (Sanchez and Feldman, 2017). These networks can be generated by various means including nucleation and release by the centrosome, microtubule fragmentation or nucleation from non-centrosοmal γ-tubulin or existing microtubules (Kuijpers and Hoogenraad, 2011). The Golgi complex is the major non-centrosomal MTOC in vertebrate cells (Rios, 2014; Wu and Akhmanova, 2017). Unlike the centrosome, the Golgi complex nucleates microtubules in a polarized manner. Accordingly, Golgi-derived microtubules are important for cell migration, perhaps by acting as a conduit for protein transport towards the leading edge (Rios, 2014).

Before neurite formation, the centrosome is highly dynamic in differentiating neurons. In the embryonic spinal cord, the centrosome is positioned at the tip of the apical process of new-born neurons that are delaminating from the neuroepithelium (Das and Storey, 2014; Kasioulis et al., 2017; Moore et al., 2020). In the chick, these neurons undergo shedding of their apical membranes through the regulated process of apical abscission, leading to acute loss of polarity (Das and Storey, 2014). However, the centrosome is retained through remodelling of sub-apical microtubules and acto-myosin constriction (Kasioulis et al., 2017), suggesting a requirement for the centrosome in the early stages of neuron polarization. Shortly after axon specification in dissociated neurons, the centrosome is deactivated (Steiss, 2010), and mature neurons are characteristically reliant on acentrosomal microtubule nucleation (Kuijpers and Hoogenraad, 2011). These studies suggest that the centrosome has a role in the initial but not the later stages of axon formation, when nucleation is likely decentralized and microtubules are tethered elsewhere, such as at the Golgi complex or cytoplasm via CAMSAPs (Meiring et al., 2020). In agreement, generation of cultured neurons with multiple centrosomes by preventing cytokinesis results in the formation of multiple neurites originating close to the centrosomes (De Anda et al., 2005). Work in dissociated neurons, cortical slices and stem cell-derived neurons also supports centrosome activity in early neurite outgrowth and axon specification (De Anda et al., 2005, 2010; Steiss, 2010; Lindhout et al., 2021). Centriole depletion in stage 2 stem-cell derived neurons results in decreased growth cone size of the immature neurites, an effect mimicked by nocodazole treatment, suggesting a microtubule-mediated mechanism (Lindhout et al., 2021). The importance of various MTOCs likely differs depending on organism. For example, the centrosome might not be as important for neurite initiation in invertebrates. *Drosophila* mutants without centrioles develop an architecturally normal nervous systems (Basto et al., 2006), and polarization and orientation of the first neurite in *Drosophila* sensory neurons is not dependent on the presence of centrioles (Pollarolo et al., 2011).

As microtubules are long, stiff filaments, the position of an MTOC within a cell can direct cell shape and trafficking (Meiring et al., 2020). In most vertebrate cells, the centrosome and Golgi are physically associated and located together. Repositioning of the centrosome–Golgi complex can rapidly shift microtubule network architecture to facilitate cellular polarity changes. An early study in mouse neuroblastoma cells showed aggregation of microtubule nucleating sites on one side of the cell around 24 h before the initiation of a neurite at the local membrane, after which microtubules appeared to be biased towards the neurite (Spiegelman et al., 1979). Since neuroblastoma cells are multicentrosomal, this suggests active centrosomal positioning might assist in channelling microtubules for neurite formation. In neurons, centrosome–Golgi complex position is non-random in relation to the location of the axonal neurite (Lefcort and Bentley, 1989; Zmuda and Rivas, 1998; De Anda et al., 2005; Zolessi et al., 2006; Andersen and Halloran, 2012; Moore et al., 2020). In rat hippocampal neurons (De Anda et al., 2005), mouse cerebellar granule neurons (Zmuda and Rivas, 1998) and grasshopper limb neurons (Lefcort and Bentley, 1989), the centrosome and Golgi cluster in the area close to the first neurite. Since in these cells this first neurite usually becomes the axon, MTOC positioning could be orienting polarization. Centrosome–Golgi complex localization is highly dynamic during neuronal differentiation (De Anda et al., 2010; Gärtner et al., 2012; Kasioulis et al., 2017; Moore et al., 2020). Live imaging has revealed that MTOC localization close to the axon base occurs after neurite initiation in hippocampal cultures (Gärtner et al., 2012), in neurons in the mouse cortex (De Anda et al., 2010), and zebrafish spinal cord (Moore et al., 2020), suggesting that proximity to the membrane is not important for initial neurite formation but might be more important for axon specification. Preceding axonal neurite initiation, the centrosome–Golgi complex has been found directly opposite the site of neurite outgrowth in spinal cord neurons in vivo (Moore et al., 2020). Likewise, the centrosome of Rohon-Beard cells is also positioned opposite the initial axon outgrowth site (Andersen and Halloran, 2012). In these cases, positioning of the centrosome opposite neurite outgrowth could favour the channelling of microtubules to the opposite side of the cell (Fig. 1).

One challenge in deciphering the influence of centrosome positioning on neurite initiation is the fact that both processes are dependent on cytoskeletal rearrangements (Tang and Marshall, 2012) and share regulators, such as PI3K and Cdc42 (Witte and Bradke, 2008), so it is unclear how active or passive centrosome positioning is in establishing polarity. It is thought that the centrioles form a structural core upon which microtubules attach and generate force. An in-vivo study of cortical neurons that used microtubule stabilization to decrease centrosome motility found aberrant centrosome localization and positioning of axon outgrowth (De Anda et al., 2010); however, it is not clear whether centrosome positioning was instructive of axon positioning or a consequence of a shared microtubule-related mechanism. Another challenge in understanding the importance of centrosome positioning is the close association between the Golgi complex and centrosome. The Golgi complex is also positioned by microtubules via mechanisms that might also involve dynein (Barr and Egerer, 2005). Thus, the relative contributions of the centrosome and Golgi complex and their positioning in neurite initiation remain unclear.

Clues to centrosomal positioning mechanisms could be found from other cell behaviours. During cell division and in wound healing, centrosomes are pulled towards the cell membrane by cortically anchored dynein–dynactin complexes (Tang and Marshall, 2012; Burakov and Nadezhdina, 2020). Also in cell division, dynein is regulated by NuMA, which is in turn regulated by the Par polarity complex (Tang and Marshall, 2012). Pushing forces exerted on microtubules might also regulate centrosome positioning (Letort et al., 2016). Dynein is dynamic in stage 2 hippocampal neurons but concentrates in the axon upon specification via interaction with kinesin-1 (Twelvetrees et al., 2016). However, the role of dynein in centrosomal positioning in neurite initiation is unclear.

## MICROTUBULE REGULATION BY OTHER CYTOSKELETAL ELEMENTS

### Actin

Actin is an important regulator of microtubules in neuronal morphogenesis, and the coordination of the two networks is paramount to neurite formation. Actin filaments are polarized polymers which, like microtubules, mainly elongate from one end (Blanchoin et al., 2014). The dynamics and arrangement of F-actin are heavily regulated in neuron polarization. Before neurite protrusion, actin undergoes polymerization-dependent reorganization into aggregates at the cell periphery (Zhang et al., 2016). During protrusion, actin-based filopodia and lamellipodia form, following which the growth cone periphery remains actin rich (Fig. 1). Actin is the major target of the Rho family GTPases, which are the key upstream regulators of neurite formation (Gonzalez-Billault et al., 2012). The roles and regulation of actin in neurite and axon development have been summarized previously and are beyond the scope of this review (Da Silva and Dotti, 2002; Luo, 2002; Kessels et al., 2011; Flynn, 2013; Sainath and Gallo, 2015).

Observations of actin–microtubule interactions in the growth cone are likely especially relevant to the engorgement phase of neurite initiation (Fig. 1). In these protrusions, actin undergoes retrograde flow (Dehmelt and Halpain, 2004). As F-actin is polymerized at the leading tip, filaments are drawn backwards. This mechanism enables membrane protrusion and facilitates a clutch system to move through the extracellular substrate. This backward current is thought to restrict microtubule advance at the leading edge. Indeed, when actin is experimentally depolymerized, microtubules advance into the peripheral growth cone (Forscher and Smith, 1988) and more neurites form (Zhang et al., 2016). At the transitional zone, contractile acto-myosin arcs constrain microtubules into the central domain (Schaefer et al., 2002). At the peripheral zone, some microtubules bend and break as they are pushed back into the transitional zone by actin retrograde flow, which enables the formation of new microtubule ends for polymerization. Attenuation of retrograde flow facilitates the protrusion of microtubules into the periphery, their polymerization guided by filopodial F-actin (Dent et al., 2007; Schaefer et al., 2008). In the neurite shaft, actin is rich at the cell cortex, forming periodic rings in the axon, and longitudinal filaments in dendrites (Xu et al., 2013). Cortical actin might also support microtubule bundles in the nascent neurite (Flynn, 2013). In neuronal differentiation, actin–microtubule crosstalk is mediated by many regulators (Dehmelt and Halpain, 2004; Flynn, 2013; Coles and Bradke, 2015; Pacheco and Gallo, 2016; Pinto-Costa and Sousa, 2021). Here, we discuss a few key factors likely to be relevant to neurite initiation (Fig. 2).

Drebrin is an F-actin side-binding protein that facilitates bundled actin formation in filopodia and has established roles in promoting process outgrowth (Dun and Chilton, 2010; Dun et al., 2012). Drebrin can also bind EB3 to promote formation of minor processes (Geraldo et al., 2008; Bazellières et al., 2012). In a recent study in cultured cortical neurons, loss of drebrin or EB3 reduced the number of peripheral dynamic microtubules and delayed neurite initiation (Poobalasingam et al., 2021). Conversely, overexpression of either protein increased the number of neurites. The authors propose that EB3 and drebrin facilitate the zippering of elongating microtubules to filopodial F-actin. This type of interaction could also be facilitated by direct crosslinking factors. Recently, Fmn2 has been revealed as a crosslinker that guides microtubules along F-actin bundles into growth cone filopodia in chick and zebrafish neurons (Kundu et al., 2021). Likewise, +TIP XMAP215 has been found to also bind actin and is proposed to align microtubule growth with F-actin in the growth cone (Slater et al., 2019).

Classical MAPs MAP1B and MAP2 also have actin-binding domains, suggesting they could fulfill a similar function in neurite initiation (Dehmelt and Halpain, 2004). MAP2C has been found to bind and bundle F-actin through its microtubule-binding domain (Roger et al., 2004). This actin binding is necessary for neurite initiation in Neuro2A cells, highlighting the importance of this interaction. In this way, classical MAPs might coordinate the rearrangement of both cytoskeletal components simultaneously.

F-actin binding proteins could also help prevent polymerization of microtubules into the cell periphery. The F-actin binding protein Capzb2 associates with tubulin to inhibit microtubule polymerization in the growth cones of cultured hippocampal neurons (Davis et al., 2009). This mechanism could temper microtubule invasion in the engorgement phase of initiation.

Motor proteins are also potential crosstalk mediators since they regulate movement of cytoskeletal elements relative to each other. For example, kinesin-12 negatively regulates axonal microtubule transport but positively regulates growth cone size and axon length (Liu et al., 2010; Dong et al., 2019). This might happen through its interactions with actin motor myosin IIB (Feng et al., 2016), which restricts microtubule advance into the growth cone (Ketschek et al., 2007).

The capture of microtubule plus ends at the actin cortex is likely a key step in neurite initiation. IQGAP is a neuronal actin-associated protein (Hedman et al., 2015) that promotes neurite formation in neuroblastoma cells (Li et al., 2005). In non-neuronal cells, IQGAP interacts with the +TIP CLIP-170 at the leading edge to regulate microtubules at their plus ends, and expression of a mutant form causes formation of extra leading edges (Fukata et al., 2002). Pausing of CLIP-170 associated microtubules in the presence of IQGAP suggests that the two regulators cooperate to enable microtubule capture at select regions of the cortex, which could facilitate the engorgement of actin-based protrusions.

Spectraplakins are +TIPs that also interact with actin (Pacheco and Gallo, 2016). The spectraplakin-family protein ACF7 aligns microtubules with F-actin, and disruption of ACF7 or the *Drosophila* homolog Shot is associated with defects in axon growth and microtubule organization (Alves-Silva et al., 2012). Another +TIP, Navigator-1, was recently reported to associate with actin and prevent microtubule depolymerization in actin-rich regions of dissociated cortical neurons (Sánchez-Huertas et al., 2020). This mechanism was found to be important for growth cone steering; however, it could also facilitate microtubule growth in neurite initiation.

### Intermediate filaments

IFs are highly stable cell-type specific cytoskeletal polymers that intimately interact with microtubules (Etienne-Manneville, 2018). Differentiated vertebrate neurons contain a dense array of IFs, which far outnumber microtubules and actin filaments and are more stable and flexible than either. Neurofilaments are the main structural IF in mature vertebrate neurons, thought to provide structural support and enhance axonal shaft calibre to improve conduction velocity (Bott and Winckler, 2020). During differentiation, neurofilaments are also required for neurite elongation and maintenance (Shea and Beermann, 1994; Lin and Szaro, 1995; Walker et al., 2001). IF protein expression patterns are highly variable between neuron types and stages of maturation (Bott and Winckler, 2020). Alongside neurofilaments, vimentin and nestin are also present in developing neurons and might help constitute a more plastic IF network in the initial stages of neurite outgrowth. Disruption of vimentin perturbs neurite formation in Neuro2A cells (Shea et al., 1993) and hippocampal cultures (Boyne et al., 1996). Likewise, depletion of IF protein peripherin by RNA-interference in PC-12 cells inhibits neurite formation (Helfland et al., 2003). Although the expression patterns and roles of individual IF proteins in neuronal differentiation are incompletely understood, these studies support a role for IFs in neurite initiation.

IFs are emerging as important regulators of microtubules, suggesting that these interactions might be important for neurite formation (Fig. 2). It should be noted that microtubules are also important regulators of IFs, as detailed in other recent reviews (Etienne-Manneville, 2018; Bott and Winckler, 2020). In the vertebrate axon, neurofilaments are proposed to form a viscoelastic hydrogel network surrounding parallel microtubules (Bott and Winckler, 2020; Prokop, 2020). This arrangement could facilitate the spacing of microtubules and the movement of microtubules and their cargo within the neuron during neurite outgrowth. Although IFs are not polar like microtubules or F-actin, their asymmetric distribution can contribute to cell polarity (Oriolo et al., 2007). In simple epithelia, IFs participate in MTOC localization and microtubule arrangement, with polarized distribution of IFs occurring before polarization of actin and microtubule networks. The exceptional stability of IFs might offer a mechanism to stabilize cell polarity during dynamic microtubule rearrangement. Live imaging of epithelial cells demonstrates that vimentin aligns with microtubules to template future microtubule growth during cell migration (Gan et al., 2016). IF proteins can directly bind tubulin to modulate microtubule number (Bocquet et al., 2009). Consistent with this, neurofilament-deficient axons display excessive microtubules, suggesting that IFs can sequester tubulin, which could form a local reserve for microtubule polymerization. In *Caenorhabditis elegans* neurons, IFs have also been reported to stabilize microtubules (Kurup et al., 2018). It is now of considerable interest to determine if similar mechanisms operate in the polarizing neuron to assist microtubule remodelling.

Although IF–microtubule interactions in neurite initiation are not well understood, various candidate factors could coordinate the two networks. Early imaging studies revealed the presence of structures bridging microtubules and neurofilaments (Hirokawa, 1982; Schnapp and Reese, 1982). This interaction could be mediated by the previously mentioned microtubule binding factors operating in complexes. For example, dynein associates with Nde1, a positive regulator of neurite formation that interacts with vimentin (Su et al., 2008). Alternatively, molecules such as plectins can directly bind microtubules and IFs (Wiche, 2021). Although plectin deficiency does not affect neurite number in dissociated neurons, neurites are longer and contain a higher proportion of acetylated (more stable) microtubules (Valencia et al., 2021). Other neuronal candidates include spektraplakins, which, in addition to coordinating microtubule–actin interactions, can interact with IFs (Röper et al., 2002; Pacheco and Gallo, 2016), placing them at the intersection of the three networks.

## CONCLUSIONS AND FUTURE DIRECTIONS

Neurite initiation is critical in establishment of neuronal polarity. Historically, the majority of neuronal polarity research has focused on how existing neurites are specified into axons and dendrites. Consequently, how neurites are initiated, and the various regulatory mechanisms involved, are rarely summarized (Dehmelt and Halpain, 2004; Flynn, 2013; Sainath and Gallo, 2015). Here, we have synthesized work from neuronal and non-neuronal cell lines, primary neurons and tissue-based studies for an up-to-date picture of microtubule regulation in neurite formation. However, many questions remain for future exploration. For example, how does MTOC positioning regulate neurite formation? How is microtubule regulation localized within the cell? And what role do IFs have?

Recent work is revealing new microtubule behaviours and modes of regulation that would be interesting to explore in neurite initiation. For instance, the influence and regulation of tubulin post-translational modifications on neuronal polarity is an emerging area of research (Kelliher et al., 2019; Moutin et al., 2020). Acetylation and trimethylation of the same tubulin residue are proposed to differentially affect microtubule mechanics in cortical neuron morphogenesis (Wei et al., 2018; Xie et al., 2021), and tubulin SUMOylation has been identified as a tubulin modification that enhances neurite growth in Neuro2A cells (Xie et al., 2021). Additionally, new roles for established microtubule regulators are being uncovered. For example, kinesin-1 has recently been found to exchange tubulin dimers along microtubule shafts leading to formation of GTP-tubulin islands and local amplification of the microtubule network at the front of polarizing cells (Andreu-Carbo et al., 2022). A new study showed that MAP6 induces microtubule coiling in developing neurons (Cuvellier et al., 2021), a conformation that could enhance resistance to compressive forces that microtubules face in process outgrowth.

Despite many extracellular signals and intracellular signalling pathways being identified that regulate microtubules in axon specification and neurite elongation (Arimura and Kaibuchi, 2007; Funahashi et al., 2014; Namba et al., 2015; Singh and Solecki, 2015; Yogev and Shen, 2017), the upstream regulators of neurite initiation specifically are not well understood. One area of future interest will be the regulation of microtubules by other cytoskeletal elements. For instance, the role of IFs in neurite formation is only partially understood and less is known about the factors that mediate IF–microtubule interactions. Septins comprise another cytoskeletal network present in neurons and important in polarizing cells (Spiliotis and Nakos, 2021). In particular, Septin 7 has been found to tag former process sites for regrowth and facilitate microtubule invasion in the initiating neurites of dorsal root ganglion neurons (Boubakar et al., 2017). It will be interesting to understand more about septin–microtubule interactions in developing neurons.

Since much research has utilized 2D cultures of dissociated cells, how de-novo neurites are formed in their native extracellular environment is unclear, particularly in vertebrates. New systems, such as stem cell-derived neuronal cultures (Lindhout et al., 2021), are facilitating detailed examination of de-novo neurite formation in a human context at the high spatial resolution afforded by cell culture. In addition, as axon growth has been found to differ in 2D and 3D culture (Santos et al., 2020), and neurite initiation is affected by the mechanical properties of the substrate (Chang et al., 2017), soft 3D culture methods might also offer a more physiologically relevant way to culture neurons. Finally, advances in in-vivo and ex-vivo approaches (Moore et al., 2020; Toro-Tapia and Das, 2020) that facilitate the study of neuronal differentiation in real time within tissue, are necessary to fully appreciate the control of neurite formation in the developing embryo.

Delineating cytoskeletal regulation throughout neurite formation will be key to understanding how morphological aberrations in neurodevelopmental disorders arise. Disruptions to several microtubule regulators in neurite formation are associated with neurodevelopmental disorders and affect neurite formation in vitro. For example, doublecortin mutations are associated with lissencephaly, a disorder of severe cortical malformation (Fry et al., 2014). Induced pluripotent stem cell-derived neuronal cells from lissencephaly patients with doublecortin mutations display stunted neurite formation (Shahsavani et al., 2018). Additionally, 14-3-3-ε overexpression, which is thought to underlie the aetiology of 17p13.3 microduplication, a genetic syndrome linked to ASD and intellectual disability (Curry et al., 2013), has been found to disrupt neurite formation in vitro by preventing microtubule invasion (Cornell et al., 2016). These studies suggest that microtubule disruption in neurite formation could contribute to human neurodevelopmental defects.

Beyond embryonic development, neurite growth facilitates nervous system repair after injury (He and Jin, 2016). However, in the adult vertebrate, while peripheral neurons have some capacity to regrow processes towards targets, central neurons usually cannot regenerate their projections, leading to devastating loss of function for patients with CNS injury. The similarity between neurite formation in developmental and regenerative contexts is unclear. As in development, microtubules are central to neurite regeneration (He and Jin, 2016; Rodemer et al., 2020). However, growth cone re-establishment in regeneration typically occurs from pre-existing processes (He and Jin, 2016), although axotomy-triggered neurite initiation has been reported in cultured hippocampal neurons (Ohara et al., 2011). Despite some important differences in microtubule regulation of mature neurons, e.g. absence of centrosomal nucleation (Kuijpers and Hoogenraad, 2011), many of the mechanisms discussed here could apply to the regenerative state and should be considered avenues for investigation. We hope that the recent advances summarized in this review will facilitate the next steps in understanding the critical process of neurite formation in both development and regeneration.

## SUPPLEMENTARY MATERIAL

Supplementary material are available at *Oxford Open Neuroscience* online.

## DATA AVAILABILITY

This publication is supported by multiple datasets that are available at locations cited in the ‘References’ section of this paper.

## CONTRIBUTIONS

V.E.H. and R.M.D. conceived and wrote the manuscript.

## CONFLICT OF INTEREST

None declared.

## Supplementary Material

suppl_data_kvac007
