# Supplementary material for: Establishing neuronal polarity: microtubule regulation during neurite initiation
Source: Oxf Open Neurosci. 2022 May 13;1:kvac007. doi: 10.1093/oons/kvac007 (PMC10913830; doi:10.1093/oons/kvac007)
Supplement: suppl_data_kvac007 [file suppl_data_kvac007.zip › kvac007_REVIEWER COMMENTS oxfnsc-2022-008.docx]

REVIEWER COMMENTS

OXFNSC-2022-008 Establishing neuronal polarity: microtubule regulation during neurite initiation

Original submission

Reviewer 1

The review describes recent advances on the role of microtubule (MT) cytoskeleton during neurite initiation process, a critical step in the establishment of neuronal polarity and nervous system development. Throughout the review, the authors describe the function of the neural microtubule cytoskeleton and the way in which its organization and dynamics are modified by microtubule-related proteins during the initiation of neurite formation.
The review summarizes how different types of MT-interacting molecules, including stabilizing and bundling proteins, motor, severing proteins, as well as microtubule-organizing centers and other cytoskeletal elements, regulate the neurite initiation process in nervous system development.
The figures included in the text are well designed and contain well-organized information


Minor comments

-Given the importance of MT cytoskeleton in neuronal development I suggest to discuss briefly the relevance of microtubule-mediated neurite initiation processes in nervous system abnormalities and human neurodevelopmental disorders.

-The authors could consider to mention the role of MT and MT interacting proteins in the Axonal initiation segment which is relevant for protein control transport to establish and maintain neuron polarity. 

-I suggest mentioning/discussing a recent paper from Lindhout FW et al 2021 (EMBO J., Centrosome-mediated microtubule remodeling during axon formation in human iPSC-derived neurons).

Reviewer 2

This is a timely and comprehensive review of microtubule roles a regulation during neurite initiation. it is well structured and written. The illustrations are excellent. My main comment is that, in addition to development, neurite initiation also occurs during regeneration of axotomized neurons after nerve lesion. For completion, it would be interesting to discuss how the principles presented here apply to such situation. A minor comment is, on page 3, about how to circumvent the issue of neurons pre-polarizing in vivo prior to neurite initiation studies in vitro. The best way to address this is to differentiate neurons from neural stem cells or even iPS cells. These are much more likely to offer a meaningful physiological context than transformed cell lines, as suggested by the authors.

Decision letter

31-Mar-2022 

Dear Dr. Das, 

Manuscript ID OXFNSC-2022-008 entitled "Establishing neuronal polarity: microtubule regulation during neurite initiation" which you submitted to the Oxford Open Neuroscience, has been reviewed.  The comments of the reviewer(s) are included at the bottom of this letter. 

The reviewer(s) have recommended publication, but also suggest some minor revisions to your manuscript.  Therefore, I invite you to respond to the reviewer(s)' comments and revise your manuscript. 

To revise your manuscript, log into https://mc.manuscriptcentral.com/oxfnsc and enter your Author Centre, where you will find your manuscript title listed under "Manuscripts with Decisions."  Under "Actions," click on "Create a Revision."  Your manuscript number has been appended to denote a revision. 

You may also click the below link to start the revision process (or continue the process if you have already started your revision) for your manuscript. If you use the below link you will not be required to login to ScholarOne Manuscripts. 

*** PLEASE NOTE: This is a two-step process. After clicking on the link, you will be directed to a webpage to confirm. *** 

https://mc.manuscriptcentral.com/oxfnsc?URL_MASK=32f53891d6aa422589b0f3c2a8941c15 

You will be unable to make your revisions on the originally submitted version of the manuscript.  Instead, revise your manuscript using a word processing program and save it on your computer.  Please also highlight the changes to your manuscript within the document by using the track changes mode in MS Word or by using bold or colored text. 

Once the revised manuscript is prepared, you can upload it and submit it through your Author Centre. 

When submitting your revised manuscript, you will be able to respond to the comments made by the reviewer(s) in the space provided.  You can use this space to document any changes you make to the original manuscript.  In order to expedite the processing of the revised manuscript, please be as specific as possible in your response to the reviewer(s). 

IMPORTANT:  Your original files are available to you when you upload your revised manuscript.  Please delete any redundant files before completing the submission. 

Because we are trying to facilitate timely publication of manuscripts submitted to the Oxford Open Neuroscience, your revised manuscript should be uploaded as soon as possible.  If it is not possible for you to submit your revision in a reasonable amount of time, we may have to consider your paper as a new submission. 

Once again, thank you for submitting your manuscript to the Oxford Open Neuroscience and I look forward to receiving your revision. 

Sincerely, 
Dr. Carlos Ibanez 
Senior Editor, Oxford Open Neuroscience 
carlos.ibanez@pku.edu.cn

Author response

We thank the referees for considering our manuscript and are delighted with their positive response. We have now addressed all referee comments. Changes to the manuscript are in green text to facilitate rapid review. 

Rewiewer 1 

-Given the importance of MT cytoskeleton in neuronal development I suggest to discuss briefly the relevance of microtubule-mediated neurite initiation processes in nervous system abnormalities and human neurodevelopmental disorders. 

Response: Thank you for your suggestion, this has been incorporated on page 1 and 13. 

-The authors could consider to mention the role of MT and MT interacting proteins in the Axonal initiation segment which is relevant for protein control transport to establish and maintain neuron polarity. 

Response: The formation of the axon initial segment is an important stage in neuronal maturation; however, since this event occurs after axon specification (Leterrier C. The Axon Initial Segment: An Updated Viewpoint. J Neurosci. 2018 Feb 28;38(9):2135-2145) and well beyond neurite initiation, we decided not to include this in the review in order to maintain focus. 

-I suggest mentioning/discussing a recent paper from Lindhout FW et al 2021 (EMBO J., Centrosome-mediated microtubule remodeling during axon formation in human iPSC-derived neurons). 

Response: Thank you for this suggestion. We discussed this paper on pages 10 and 13. We have now also included this paper in our discussion of in vitro model systems to investigate neurite initiation on page 2. 

Reviewer 2 

- My main comment is that, in addition to development, neurite initiation also occurs during regeneration of axotomized neurons after nerve lesion. For completion, it would be interesting to discuss how the principles presented here apply to such situation. 

Response: Thank you for your suggestion. We have updated the discussion to include this topic. 

- A minor comment is, on page 3, about how to circumvent the issue of neurons pre-polarizing in vivo prior to neurite initiation studies in vitro. The best way to address this is to differentiate neurons from neural stem cells or even iPS cells. These are much more likely to offer a meaningful physiological context than transformed cell lines, as suggested by the authors. 

Response: Thank you for pointing this out. We have discussed iPS cell derived neurons on page 10 and 13 and have now also amended our discussion of in vitro models to study neurite initiation on page 2.

Revised submission

Reviewer 1

I consider that the authors have satisfactory addressed all my comments and now it is suitable for publication.

Reviewer 2

This piece can now be accepted for publication

Decision letter

02-May-2022 

Dear Dr. Das, 

It is a pleasure to accept your revised manuscript entitled "Establishing neuronal polarity: microtubule regulation during neurite initiation" in its current form for publication in the Oxford Open Neuroscience.  
Please note that this journal operates with transparent peer review. This means that  the full peer review history of your article will publish online alongside your article. This includes reviewer comments, editor decision letters, and your author responses. 

Thank you for your fine contribution.  On behalf of the Editors of the Oxford Open Neuroscience, we look forward to your continued contributions to the Journal. 

Sincerely, 

Dr. Carlos Ibanez 
Senior Editor, Oxford Open Neuroscience 
carlos.ibanez@pku.edu.cn
